# An unexpected switch in peptide binding mode: from simulation to substrate specificity

**DOI:** 10.1080/07391102.2017.1407674

**Published:** 2018-01-31

**Authors:** Ursula Kahler, Julian E. Fuchs, Peter Goettig, Klaus R. Liedl

**Affiliations:** a Faculty of Chemistry and Pharmacy, Institute of General, Inorganic and Theoretical Chemistry, University Innsbruck, Innrain 82, InnsbruckA-6020, Austria; b Division of Structural Biology, Department of Molecular Biology, University of Salzburg, Billrothstrasse 11, SalzburgA-5020, Austria

**Keywords:** chymotrypsin-like serine protease, specificity subsites, peptide recognition, *in silico* mutation, drug design

## Abstract

A ten microsecond molecular dynamics simulation of a kallikrein-related peptidase 7 peptide complex revealed an unexpected change in binding mode. After more than two microseconds unrestrained sampling we observe a spontaneous transition of the binding pose including a 180° rotation around the P1 residue. Subsequently, the substrate peptide occupies the prime side region rather than the cognate non-prime side in a stable conformation. We characterize the unexpected binding mode in terms of contacts, solvent-accessible surface area, molecular interactions and energetic properties. We compare the new pose to inhibitor-bound structures of kallikreins with occupied prime side and find that a similar orientation is adopted. Finally, we apply *in silico* mutagenesis based on the alternative peptide binding position to explore the prime side specificity of kallikrein-related peptidase 7 and compare it to available experimental data. Our study provides the first microsecond time scale simulation data on a kallikrein protease and shows previously unexplored prime side interactions. Therefore, we expect our study to advance the rational design of inhibitors targeting kallikrein-related peptidase 7, an emerging drug target involved in several skin diseases as well as cancer.

## 1. Introduction

Kallikrein-related peptidase 7 (KLK7, hK7) is a chymotrypsin-like serine protease and part of the kallikrein family formed by 15 homologous proteolytic enzymes (Yousef, Scorilas, Magklara, Soosaipillai, & Diamandis, 2000) that appears to be a specific feature of mammals (Lundwall, 2013). The enzyme is mostly expressed in the skin and is crucial for skin homeostasis (Brattsand, Stefansson, Lundh, Haasum, & Egelrud, 2005). Thus, KLK7 has been linked to several skin disorders including dermatitis (Komatsu et al., 2007; Yamasaki et al., 2007), psoriasis (Ekholm & Egelrud, 1999) and the Netherton syndrome (Descargues et al., 2005). The molecular link appears to be the KLK7-mediated degradation of extracellular and intracellular proteins involved in the cellular structure of the *stratum corneum*, which is required for desquamation, the removal of outer skin layers (Caubet et al., 2004). Moreover, KLK7 cleaves insulin and is coexpressed with the serpin vaspin in mammalian pancreatic β-cells. Failing KLK7 regulation by vaspin is a putative factor of diabetes (Heiker et al., 2013). Additionally, over-expression of KLK7 has been described as a potential route for metastasis in several cancer types (Dong, Kaushal, Brattsand, Nicklin, & Clements, 2003; Johnson, Ramani, Hennings, & Haun, 2007; Rezze, Fregnani, Duprat, & Landman, 2011; Talieri et al., 2009). These key functions in fundamental cellular processes turn KLK7 into a promising drug target (Prassas, Eissa, Poda, & Diamandis, 2015).

In addition to inhibition by several endogenous proteins, e.g. LEKTI (Deraison et al., 2007) and SPINK6 (Meyer-Hoffert et al., 2010), heavy metal ions regulate enzyme activity (Franzke, Baici, Bartels, Christophers, & Wiedow, 1996). Several synthetic small molecules have been identified as KLK7 inhibitors. The ChEMBL bioactivity database (Bento et al., 2014) currently lists 64 unique compounds. This includes diverse chemical classes ranging from natural isocoumarins (Teixeira et al., 2011), isomannide-based peptidomimetics (Freitas et al., 2012; Hoelz et al., 2016), 1,2,4-triazoles (Tan et al., 2013), pyrido-imidazodiazepinones (Arama et al., 2015) to recently reported halomethyl-based suicide inhibitors (Tan et al., 2015) and cyclic peptide inhibitors based on sunflower trypsin inhibitor-1 (de Veer, Wang, Harris, Craik, & Swedberg, 2015). A 3D-quantitative structure-activity relationship study investigated coumarin-derived KLK7 inhibitors and proposed their binding mode by docking (Zheng et al., 2017). Natural and synthetic inhibitors of the kallikrein family and their structural context have been reviewed in 2010 (Goettig, Magdolen, & Brandstetter, 2010).

To date six crystal structures of KLK7 provide insights into molecular mechanisms of enzymatic function and inhibition. The chymotrypsin-like serine protease has been characterized as ligand-free enzyme (PDB: 3BSQ (Fernández et al., 2008)) as well as in complex with two different peptide-based covalent inhibitors (PDB: 2QXG, 2QXH, 2QXI, 2QXJ (Debela et al., 2007)) and one non-covalent inhibitor (PDB: 5FAH (Maibaum et al., 2016)). Asn189 at the bottom of the S1 specificity pocket accounts for the chymotryptic specificity of KLK7 (Hansson et al., 1994; Skytt, Stromqvist, & Egelrud, 1995). Hydrophobicity of the larger S1 pocket is enhanced by presence of Ala-190 (Debela et al., 2008). Beyond the S1 pocket, specificity of KLK7 has been characterized for the non-prime region of the binding site that shows non-specific binding especially in case of S3 and S4 (Debela et al., 2006). Recently, proteomics and FRET-based approaches have been used to characterize the substrate specificity of KLK7 also in the prime side region (Oliveira et al., 2015; Yu, Prassas, Dimitromanolakis, & Diamandis, 2015). In addition to static binding site properties flexibility of adjacent loop regions, especially the 99-loop, has been described as crucial for enzymatic function of KLKs (Skala et al., 2014).

To aid drug design efforts targeting KLK7 we performed a large time scale molecular dynamics simulation of the enzyme in complex with a substrate peptide. The sampling time of 10 μs allowed us to recover a rare event from the simulation trajectory, a spontaneous transition of the binding region from the non-prime region to the prime side. Based on the recovered structural ensemble and protease-peptide interactions, we propose a first structural model for prime side binding of a peptide in KLK7 and further apply *in silico* mutagenesis to explore substrate specificity in this scarcely characterized binding site region. As protease substrates provide guidance for the development of drug-like inhibitors (Drag & Salvesen, 2010; Fairlie et al., 2000), we expect our analyses highly valuable for further structure-based design efforts targeting the kallikrein family.

## 2. Methods

### 2.1. Structure preparation and visualization

**Figure 1.**
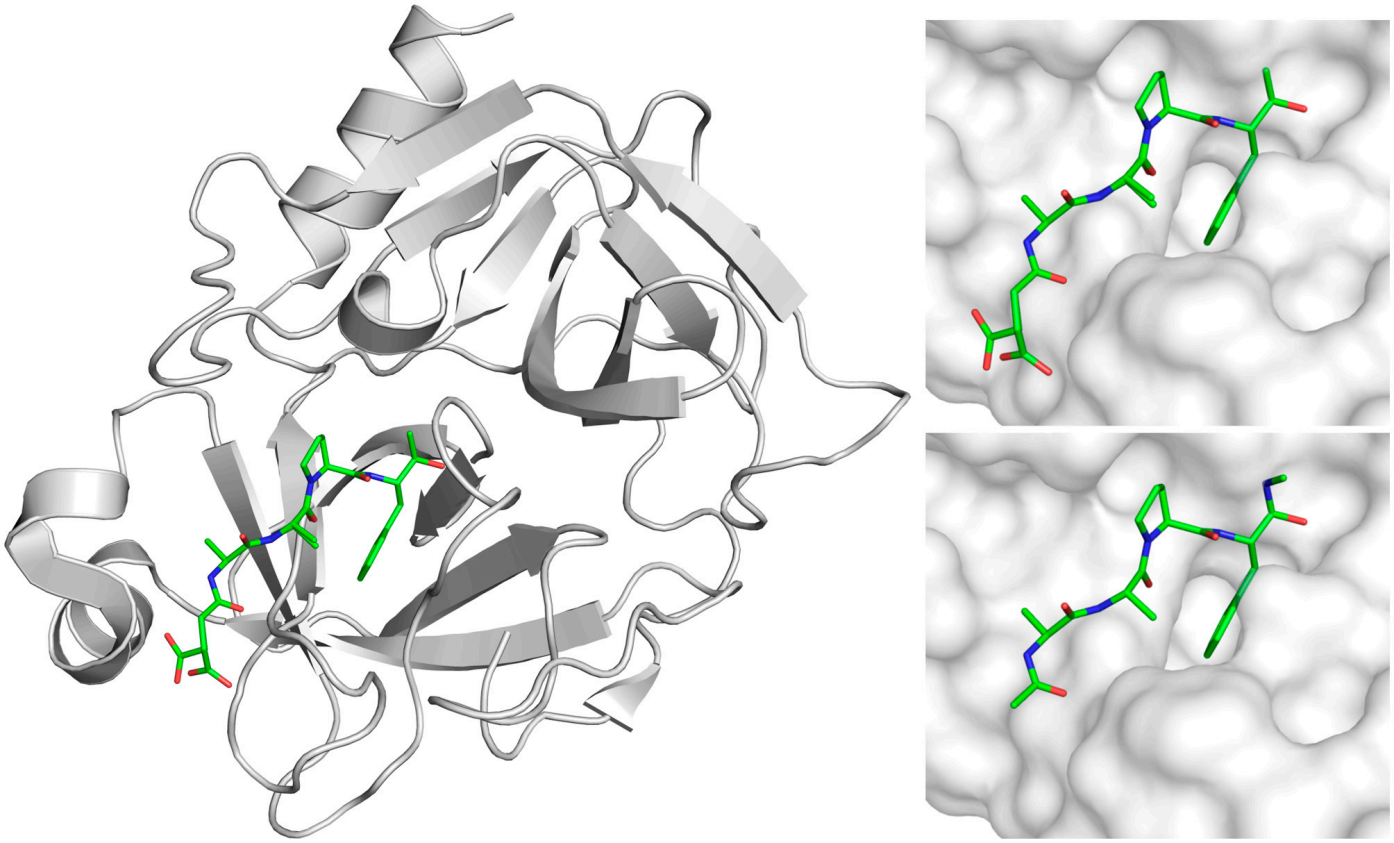
The KLK7 X-ray structure 2QXI (left) with the covalently bound inhibitor suc-Ala-Ala-Pro-Phe-chloromethyl ketone (top right) was modified to yield peptide model Ac-Ala-Ala-Pro-Phe-N-methyl (bottom right) for the *in silico* approaches.

The presented molecular dynamics simulation study was based on the crystal structure of KLK7 in complex with an active site bound succinyl-AAPF-chloromethylketone ligand at 1.0 Å resolution (PDB: 2QXI [Debela et al., 2007]). We generally used the A conformation, broke the bonds to Ser-195 and His-57 of the ligand and modified its tail groups (Figure 1). Our system set-up represents a non-covalent protease-substrate complex of KLK7 in complex with an AAPF tetrapeptide that is capped on both terminals with an acetyl and an N-methyl group, respectively. We refer to the ligand residues later on as Ala-1, Ala-2, Pro-3 and Phe-4. The introduced cap groups were energy minimized after adding hydrogens to the system according to physiological pH using protonate3D (Labute, 2009). Hence, the system consists of 224 KLK7 residues (sequence can be found in the SI) plus the capped four-residue peptide ligand. All structures were visualized using pymol (PyMOL, 2015).

### 2.2. Molecular dynamics simulations

The system was described using the Amber force field 99SB (Hornak et al., 2006) with ILDN corrections (Lindorff-Larsen et al., 2010) within Amber12 (Case et al., 2012). The system was soaked into a truncated octahedral water box of explicit TIP3P water molecules with a minimum wall distance of 12.0 Å in addition to water molecules resolved in the crystal structure (Jorgensen, Chandrasekhar, Madura, Impey, & Klein, 1983). The box net charge of +12 was neutralized using a uniform neutralizing plasma for Particle Mesh Ewald simulations (Darden, York, & Pedersen, 1993). Simulations were performed at 300.0 K and 1 bar using the CUDA implementation of pmemd (Salomon-Ferrer, Götz, Poole, Le Grand, & Walker, 2013) applying a non-bonded cut-off of 8.0 Å. After performing an in-house developed extensive equilibration protocol involving several heating and cooling steps (Fuchs et al., 2012), we performed 10 μs of unrestrained sampling using a 2.0 fs time step enabled via SHAKE algorithm on hydrogen atoms (Ciccotti & Ryckaert, 1986). Snapshots were saved to trajectory every 10,000 steps or equivalent 20 ps for further analysis, thus resulting in a conformational ensemble of 500,000 snapshots.

### 2.3. Analysis of molecular dynamics simulations

Trajectories were analysed using cpptraj from AmberTools (Roe & Cheatham, 2012). We calculated root mean square distances (RMSDs) of Cα atoms after a global alignment of all Cα atoms of the protein to the structure after equilibration to assess stability of our simulation. The peptide RMSD was calculated following the same alignment to the protein and thus explicitly contains movements of the peptide relative to KLK7. 2D-RMSD plots of protein and peptide Cα atoms were generated analogously for 1,000 equal-spaced snapshots to assess similarities of occurring conformations over simulation time. These plots pairwisely compare frames of the simulation with each other and help to elucidate the presence of visited and revisited conformations. To further check the stability of the simulation and to make sure the protein fold is retained, the energy evolution and the total secondary structure content were recorded. Flexible protein regions were identified by calculation of b-factors of the Cα atoms after alignment on all protein Cα atoms (Fuchs et al., 2015).

After globally aligning to the protein Cα atoms, changes in the peptide and protein conformations were assessed via a hierarchical agglomerative clustering (Shao, Tanner, Thompson, & Cheatham, 2007) of all snapshots to 10 clusters each. We used the peptide backbone atom RMSD as cluster criterion for peptide binding poses. Protein conformations were assessed for the binding site region using the same algorithm. The binding site region includes all residues that are within 4.0 Å of the peptide in any frame of the simulation (corresponding residues: 41, 57, 58, 96, 97, 99, 149, 151, 190–196, 213–218, 220, 220A). Representative structures for subsequent visual inspection and their respective fractional occupancy were extracted.

Additionally, we characterized hydrogen bonding between protein and peptide using cpptraj default criteria for angles and distances. We applied an occupancy cut-off of minimum 5.0% for the analysis of recovered polar contacts. Furthermore, we calculated residue-wise non-bonded energies between peptide and protein with the linear interaction energy (LIE) approach implemented in cpptraj using default cut-offs. The free energy differences of the found peptide poses were estimated using the MM-PBSA approach available via MMPBSA.py from AmberTools (Miller et al., 2012). No entropy correction was applied and the ion concentration was set to 0 in agreement with the simulation set-up.

Contact residues were extracted from the trajectory by applying a maximum distance between heavy atoms of 4.0 Å. Native contacts were extracted from the equilibrated structure. Solvent-accessible surface area (SASA) values were extracted for the peptide residues using the LCPO algorithm (Weiser, Shenkin, & Still, 1999).

An average structure of the peptide after the observed conformational transition was extracted from the last 7 μs simulation time by averaging heavy atom positions. Subsequently, a prime side peptide P1′–P4′ AAAA was constructed ensuring maximum shape overlap with the average peptide position from the simulation. A residue-wise energy minimization using the Amber12:EHT force field (Case et al., 2012) was performed for the prime side residues using MOE (MOE, 2014). Inhibitor-bound kallikrein structures from the PDB were aligned to the resulting average structure using pymol.

### 2.4. *In silico* mutagenesis

We used the ‘Residue Scan’ interface in MOE to perform *in silico* mutagenesis experiments of the peptide complex. We mutated all P1′–P4′ in the peptide ligand from alanine to all other 19 natural amino acids using MOE’s default settings and extracted estimations for the binding affinity of 19*4 single point mutants. Additionally, we performed an ensemble refinement of the enumerated prime side peptides to include conformational flexibility. All mutagenesis calculations were performed using the Amber12:EHT force field. Affinities represent a Boltzmann-weighted average of the affinities within the ensemble.

## 3. Results

### 3.1. Characterization of peptide binding switch

**Figure 2.**
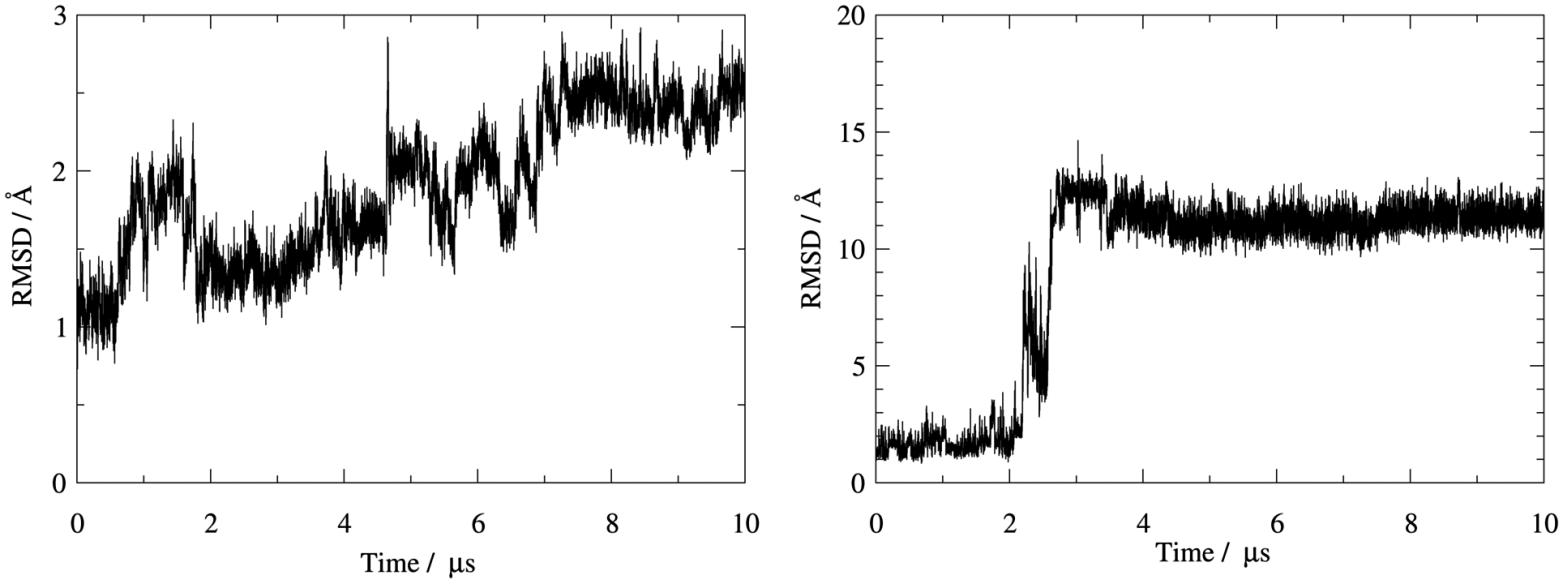
The 1D-RMSD plots of KLK7 (left) and of the ligand (right) with different scaling of y-axis show that while the protease itself stays rather stable during the simulation the peptide undergoes major rearrangements.

**Figure 3.**
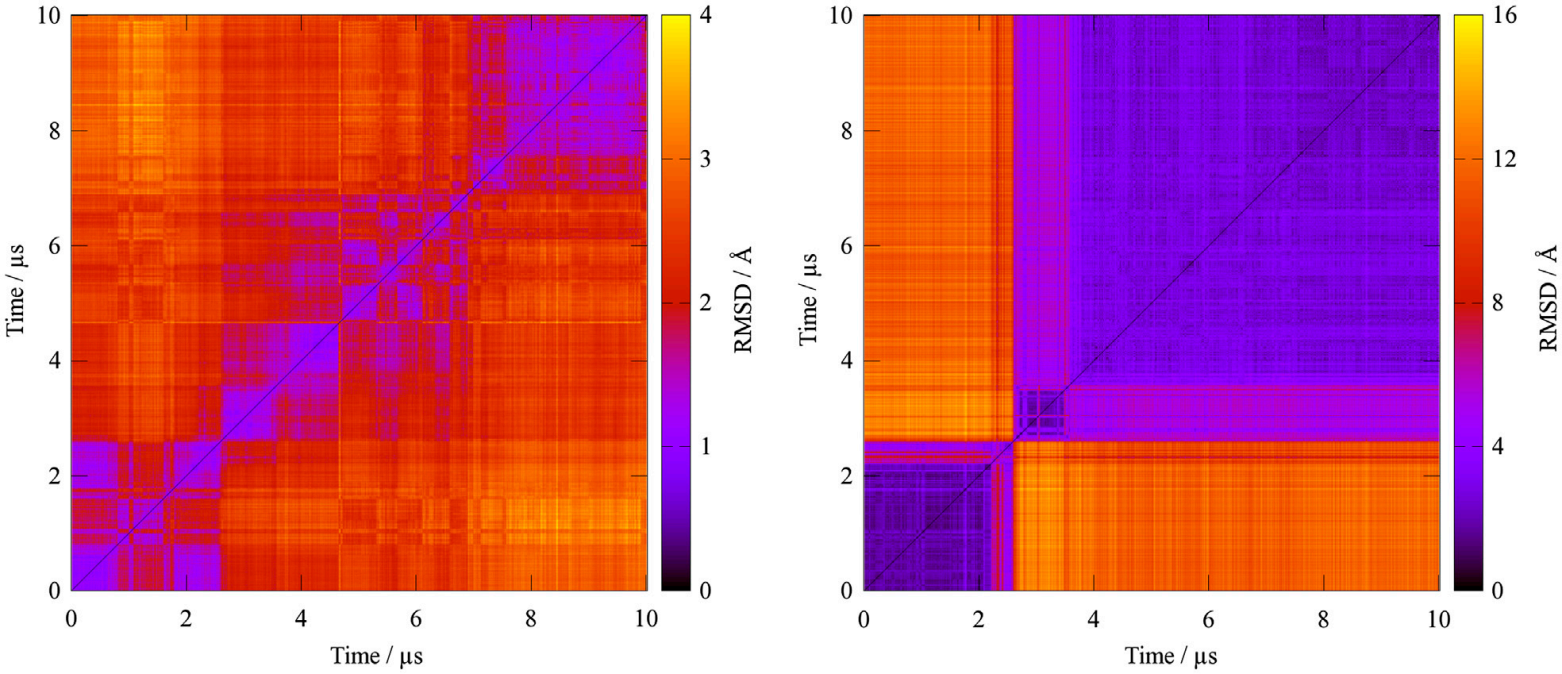
While the ligand experiences large structural shifts during the simulation (right), the conformational changes of KLK7 (left) remain small as the 2D-RMSD shows (note the different scaling of the heat map).

Molecular dynamics simulations yielded stable trajectories over the long time scale of 10 μs with a Cα-RMSD for protein atoms below 3.0 Å to the equilibrated structure (Figure 2). By contrast, the bound peptide is less stable as indicated from the peptide backbone RMSD after alignment to the protein (Figure 2). Here, RMSD values exceeding 10.0 Å occur after a conformational transition between 2.0 and 3.0 μs simulation time. In between, a meta-stable state is occupied over several hundred nanoseconds with an RMSD of around 7.0 Å compared to the starting structure. A 2D-RMSD analysis further illustrates the stability of the protease versus the extreme conformational transition observed for the bound peptide (Figure 3). Three distinct ligand conformations can be identified. The total energy of the simulated system is almost unchanged upon the conformational transition (Supplementary Figure S1), thus indicating that the change in binding mode represents an accessible state rather than an artificial drift of the system.

**Figure 4.**
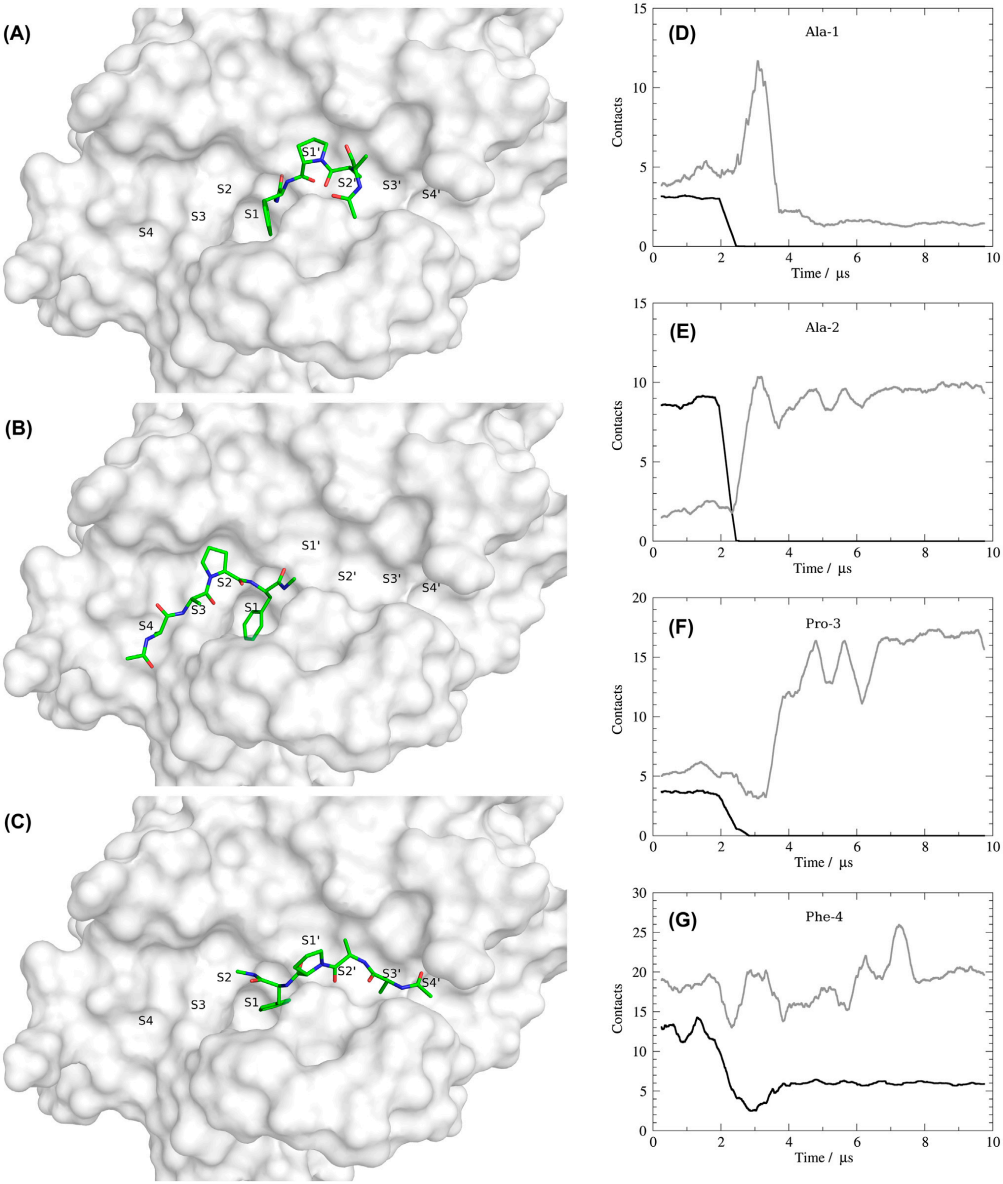
Representative structures of the three ligand-based clusters show the transition from a non-prime side bound peptide (B) over an intermediate (C) to the prime side bound and most-populated state (A). The residue-wise contact plots of the ligand residues Ala-1, Ala-2, Pro-3 and Phe-4 (D–G) show the number of native contacts in black and newly formed contacts in grey (running average of 100 ns).

To further characterize the unexpected change in the binding mode of the peptide, we clustered the trajectory into ten distinct states according to the RMSD of peptide atoms after alignment to KLK7 (Supplementary Figure S2). Only three of the ten peptide states show fractional occupancies larger than one percent and representative structures are given in Figure 4A–C as an overlay with the equilibrated protein structure. Figure 4B shows the peptide conformation in an orientation similar to the starting structure occupying the non-prime binding site region. This conformation is occupied in 24.5% of the trajectory, corresponding to the time frame until the transition is observed after around 2.5 μs simulation time. The final binding pose of the peptide is identified as dominant cluster with 65.6% occupancy with a representative structure shown in Figure 4A. A complete switch of the binding mode from the non-prime side (left) to the prime side (right) is observed, while the P1 residue (Phe-4) remains in place. Therefore, we observe a rotation of approximately 180° around an axis perpendicular to the S1 pocket, resulting in reversed peptide backbone with respect to the canonical substrate binding. The minor third cluster representative depicted in Figure 4C (occupancy 7.5%) shows an intermediate state of the peptide tail between non-prime and prime side and represents the transition between both stable states. This state is only populated between 2.5 and 3.5 μs simulation time and shows a broader distribution of RMSD values.

### 3.2. Protein-peptide contacts

These state transitions are also reflected via major changes in protein-peptide contacts (see Figure 4D–G for a residue-wise contact analysis for the four peptide amino acids). Whereas all native contacts of the three N-terminal peptide residues (AAP) are lost after 3.0 μs, the S1-bound Phe residue keeps some of its native contacts throughout the whole simulation time. Between 2.0 and 3.0 μs all three N-terminal residues form several contacts. In case of the N-terminal Ala-1 they are broken again after the transition is completed, for Ala-2 and Pro-3 they remain intact. Especially for Pro-3 the total amount of contacts is significantly increased upon inversion of the binding mode.

Similar trends are observed for residue-wise SASAs (Table 1). Whilst Ala-2 and Phe-3 show a net loss in SASAs and hence are more buried after the conformational change, Ala-1 and Phe-4 show an increase. Overall, a slight increase in total SASA is observed. Nevertheless, Pro-2 appears especially stabilized after switching to the non-prime region.

**Table 1.** Residue-wise solvent-accessible surface area of the bound peptide: SASA values are presented as average and standard deviation in brackets for the time frames before (0.0–2.0 μs) and after the observed conformational change (3.0–10.0 μs). While Ala-1 and Phe-4 are more exposed after the transition, especially Pro-3 appears more buried.

SASA/Å²	Ala-1	Ala-2	Pro-3	Phe-4
0.0–2.0 μs	53.8 (9.2)	23.5 (6.8)	51.9 (8.0)	30.6 (11.4)
3.0–10.0 μs	85.9 (20.1)	17.0 (9.6)	19.6 (23.2)	60.1 (20.9)
Difference	+32.1	−6.4	−32.3	+29.5

On protease residue level Ala-1 is tightly bound to the S4 pocket contacting residues 215–218 during the first 2 μs. After transition to the prime side, a larger variety of residues is contacted including His-41 and Asn-192. Over the first 2 μs simulation time Ala-2 is consistently bound to Trp-215, Gly-216 and Phe-218. Similar strong interactions are established after switching to the prime side by contacts to His-41, Phe-151, Asn-192 and Gly-193. Pro-3 interacts with the hydrophobic S2 pocket formed by His-57, His-99, Ser-214 and Trp-215. These tight interactions are broken when switching to the prime side and forming new contacts to several residues including His-41, Cys-58, Asn-192, Gly-193 and Ser-195. Interactions between Phe-4 and the S1 pocket are partially present over the whole simulation time (Figure 4G). Main anchor points are the catalytic residues His-57 and Ser-195 as well as Ala-190, Cys-191 and Trp-215.

No polar contacts (hydrogen bonds) to the protein are formed by Ala-1. Before the conformational transition, the backbone of Ala-2 interacts with the backbone of Gly-216 on the bottom of the binding groove in a short antiparallel β-sheet. After switching to the prime side, the side-chain of Asn-173 acts as hydrogen bond donor to the carbonyl of Ala-227 with a total occupancy of 46%. Upon switching to the prime side the carbonyl group of Pro-3 establishes hydrogen bonding with the protease via the backbone of Gly-193 and the side-chain of Ser-195 (total occupancies 42 and 9% respectively). The P1 residue Phe-4 forms consistent polar contacts throughout the whole trajectory by contacting the side-chains of the catalytic residues His-57 and Ser-195 via its backbone carbonyl (total occupancies 62% and 6%, respectively).

### 3.3. Interaction energies

These changes in molecular interactions are further reflected in intermolecular energy contributions as electrostatic interactions (Table 2) and nonpolar van der Waals interactions (Table 3). All residue-wise electrostatic interaction energies between protease and bound peptide are increased by the transition to the prime side. Pro-3 is especially involved and shows a gain of on average −4.3 kcal/mol in electrostatic interactions implied by the aforementioned formation of hydrogen bonds. Differences in van der Waals energies are directly linked to the changes in SASA. Ala-1 and Phe-4 are more solvent-exposed after switching to the prime side and accordingly show weaker van der Waals interactions with the receptor (+4.0 and +3.0 kcal/mol, respectively). These losses are partially compensated for by additional contacts formed by Ala-2 and Pro-3 leading to a gain in van der Waals interactions (−0.7 and −1.3 kcal/mol, respectively). In total, we observe a loss in van der Waals interactions between protease and peptide by switching to the prime side that is compensated by additional electrostatic interactions. Overall, the observed transition appears slightly favourable for the tetrapeptide (−0.7 kcal/mol).

**Table 2.** Residue-wise electrostatic interaction energies between KLK7 and the bound peptide. Electrostatic terms from the applied force field have been extracted to investigate energetic consequences of the observed transition in the binding mode and are presented as average and standard deviation in brackets. All residues show a net gain of electrostatic interactions when comparing the native binding pose (0.0–2.0 μs) with the inverted mode (3.0–10.0 μs).

*E*_el_/kcal/mol	Ala-1	Ala-2	Pro-3	Phe-4
0.0–2.0 μs	−5.0 (1.7)	−5.8 (1.9)	−1.7 (1.9)	−16.2 (5.0)
3.0–10.0 μs	−5.6 (4.2)	−6.2 (2.8)	−6.0 (2.7)	−16.6 (5.6)
Difference	−0.7	−0.4	−4.3	−0.3

**Table 3.** Residue-wise van der Waals interaction energies between KLK7 and the bound peptide. Average and standard deviation in brackets are given for van der Waals interactions extracted from the applied force field. Ala-1 and Phe-4 lose van der Waals interactions by switching the binding mode between 2.0 and 3.0 μs, while especially Pro-3 gains additional van der Waals interactions.

*E*_vdW_/kcal/mol	Ala-1	Ala-2	Pro-3	Phe-4
0.0–2.0 μs	−6.4 (1.0)	−5.5 (1.2)	−8.7 (0.9)	−18.5 (2.3)
3.0–10.0 μs	−2.4 (1.8)	−6.2 (1.4)	−10.0 (2.3)	−15.5 (2.5)
Difference	+4.0	−0.7	−1.3	+3.0

MM-PBSA results agree with a slight gain in binding energy upon the binding site switch of the peptide to the prime side. While MM-PBSA calculations predict a weak but favourable binding free energy of −1.14 kcal/mol when averaging over the whole 10 μs trajectory, we observe an energetic difference between both binding modes. Averaging over the first 2 μs of the trajectory with the peptide ligand bound to the non-prime side, we calculate a very weak binding free energy of −0.43 kcal/mol. Averaging over snapshots after the switch to the non-prime side (last 7 μs simulation time), we calculate a gain in binding free energy to −2.54 kcal/mol. As already shown above using the LIE approach, we find a difference in driving forces for the two binding modes. Whereas non-prime side binding is mostly driven by van der Waals interactions (ratio approximately 2:1), the prime side binding mode additionally provides attractive electrostatic interactions (ratio close to 1:1).

### 3.4. Conformational changes in KLK7

Not only the peptide itself is affected by the observed change in binding mode, we also observe slight conformational changes in the receptor. By clustering the trajectory according to the RMSD of binding site heavy atoms to three clusters, we recovered three conformations that are occupied with more than five percent. In agreement with 1D- and 2D-RMSD plots of the protein, we do not observe a concerted conformational transition in the protein, when the bound peptide switches its binding mode. The protease accepts the observed conformational transition of the binding partner in a static way. Therefore, the dominant cluster is close to the starting structure and extends beyond 6 μs simulation time. Afterwards, we observe a response of the S4 binding site region including residues 217–220. The absence of the bound peptide leads to a rearrangement of the region conveying a shallower shape of the binding groove. The cis peptide conformation present in the experimental structure between Phe-218 and Pro-219 is retained over the simulation time. Nevertheless, the prime side regions remain virtually unaffected and closely resemble the starting structure even in presence of the tetrapeptide.

Overall, no large conformational changes occur in the protein receptor during the simulation. The evolution of the secondary structure content confirms that no unfolding events take place (Supplementary Figure S3). Most of the protein core is very rigid, only peripheral loop regions and the C-terminus are flexible (Supplementary Figure S4), explaining protein RMSD values of about 3 Å.

### 3.5. Prime side binding model

**Figure 5.**
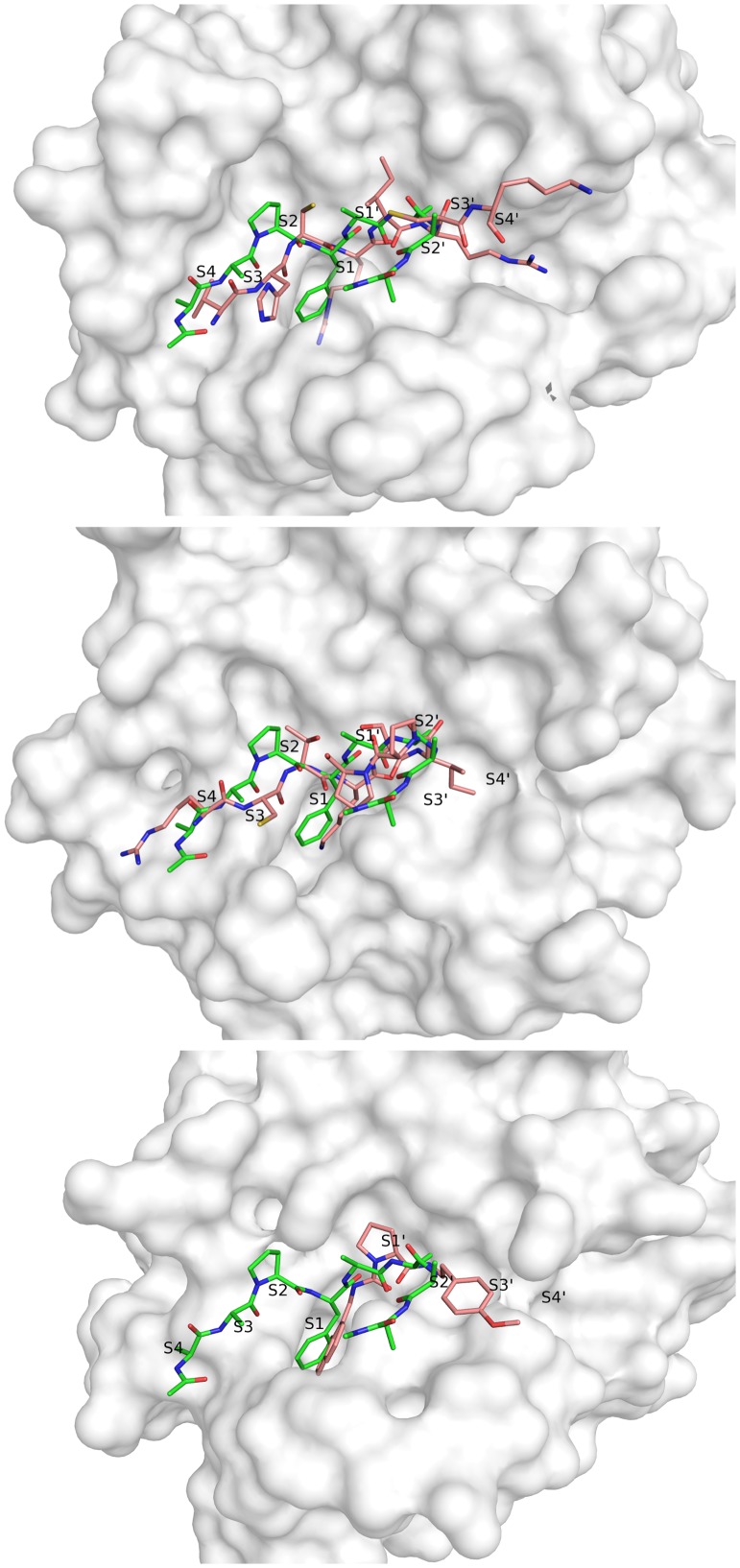
The peptide (modelled based on simulation data and shown in green) is compared to the P4–P4′ residues of the two peptidic inhibitors extracted from structure 1HIA (top) and structure 4K8Y (middle) and to the prime side bound inhibitor from structure 5FAH (bottom) in pink.

Based on the structural ensemble formed by the peptide ligand over the last 7 μs simulation time, we extracted an average structure for the reversed backbone trace, constructed and energy-minimized an AAAA tetrapeptide occupying the non-prime region (P1′–P4′). The resulting model was found to be structurally very similar to the dominant conformation recovered from ligand-based clustering and was subsequently compared to a structure of KLK7 with a prime side small-molecule inhibitor (Maibaum et al., 2016) and to two available crystal structures of kallikreins in complex with substrate-like protein inhibitors (Figure 5). The latter complexes comprise a crystal structure of KLK1 in complex with hirustasin with a P4–P4′ sequence of VHCR-IRCK (PDB: 1HIA [Mittl et al., 1997]) and a co-crystal structure of KLK4 and sunflower trypsin inhibitor SFTI-1 with P4–P4′ sequence RCTK-SIPP (PDB: 4K8Y [Riley et al., 2016]).

We find major overlap between our structural model and all crystal structures. Especially P1′ and P2′ residues are found to be in best agreement with crystal structure data. For these residues, side-chain exit vectors point in exactly the same directions as found for the substrate-like inhibitor structures and the peptidomimetic part of the small-molecule inhibitor shows a high analogy in these regions too. Residues P3′ and P4′ are less well-defined in our modelled peptide and also show broader variation within the conformational ensemble recovered from the simulation trajectory. Likewise, these residues show elevated B-factors in the KLK1/hirustasin structure. The KLK4/SFTI-1 structure shows two proline residues at those positions, which increase rigidity. Additionally, both inhibitors contain disulfide bonds close to the catalytic centre, which restrain the substrate-like conformation (P2–P18′ and P3′–P20′ in hirustasin and P3–P6′ in SFTI-1). For the outer residues P3′ and P4′ our modelled binding mode lies in between the backbone conformations adopted by the protein inhibitors.

To investigate residue preferences of KLK7 in the non-prime region we scanned all 20 amino acids at the P1′ to P4′ positions using the modelled complex as starting structure for an ensemble refinement. For all pockets we find broad distributions of predicted binding affinities with a maximum difference between most favourable and unfavourable amino acid of 2.2 kcal/mol (Table S1). This finding already points towards promiscuous binding without preference for particular amino acids. Nevertheless, we observe energy trends for each of the prime side positions. At P1′ charged amino acids are preferred with 1 kcal/mol over small hydrophobic residues like Ala. Similar trends are observed for P2′ where charged side-chains and hydrogen bond donor functions appear favoured. Energetic differences for exchanges at P3′ and P4′ are smaller, which agrees with the observed flexibility of the peptide backbone. All three positively charged amino acids (Arg > His > Lys) are preferred at P3′, whereas both bulky and positively charged residues are favoured at P4′ (Arg > Leu > Lys > Trp). This overall preference of hydrophilic residues again indicates solvent exposure of the non-prime side in KLK7.

## 4. Discussion

The surprising switch of the non-prime side residues of the tetrapeptide model bound to the prime side of KLK7 in the molecular dynamics simulation was facilitated by removal of the covalent bonds of the chloromethyl ketone to His-57 and Ser-195, present in the PDB file 2QXI. The interaction of the Phe side chain with the overall hydrophobic S1 pocket was sufficient to fix the P1 residue and to allow the 180° rotation of the P2-P4 stretch. A synthesized molecule capped with an N-terminal acetyl and a C-terminal N-methyl group for KLK7 binding studies could answer the question, whether the reverse binding to the prime side is the preferred binding mode.

Intriguingly, in special cases of physiological interactions forward and reverse binding of polypeptides appears to be equally possible, as observed in structures of the *E. coli* chaperone DnaK (Zahn et al., 2013). Reverse binding of natural peptide backbones to proteases is rarely observed, with some remarkable exceptions, such as the complex of X-linked inhibitor of apoptosis protein (XIAP) and caspase-3 (Riedl et al., 2001). Another example is the family of anti-coagulant thrombin inhibitors, the mosquito anophelins, which binds in this mode as well (Figueiredo et al., 2012). An engineered Fab antibody fragment that inhibits trypsin-like matriptase binds with the hypervariable loop H3 partially in the reversed backbone mode, e.g. P1 in S1, but P2′ in S2 and P3′ in S3 (Farady, Egea, Schneider, Darragh, & Craik, 2008). Furthermore, small-ubiquitin.like modifiers (SUMO) can bind peptides in parallel and antiparallel orientation. A study that assessed the stability of these complexes observed an inversion of the binding mode during MD simulations (Jardin, Horn, & Sticht, 2015). In the field of drug design reversed backbone peptides with L-amino acids do not play a significant role, since they are rapidly degraded by proteases, in contrast to other peptidomimetics, such as retro-inverso peptides, which are composed of D-amino acids (Fischer, 2003).

While a comparison of peptide substrate data in a positional scanning library shows only the non-prime side preferences for P4 to P1, such as -/-/yl/Yma (Debela et al., 2006), cleavage sites in selected natural substrates exhibit a higher frequency of positively charged residues at prime side positions P1′–P4′ (Debela et al., 2008) similar to our *in silico* mutational screening results. In 17 cleavages of the MEROPS specificity matrix for KLK7 (S01.300) a broader distribution with 8 different amino acids on average occurs at all positions P1′–P4′, exhibiting mostly smaller hydrophobic and basic residues and resulting in the ideal sequence i/-/-/fry†-/v/g/g (Rawlings, Barrett, & Bateman, 2012). This data might be slightly biased by the cleavage of the predominantly tryptic propeptide substrates from the studies on the KLK activation cascades, with the consensus sequence R-IVGG (Yoon et al., 2007, 2009). Cleavage data of fluorogenic peptides confirms the previously reported high preference in P1 for Tyr over Phe, while Arg occurs in less than 10% of cleavages and Lys or Ala not at all, whereas the specificity of the other subsites on the non-prime and prime side seems relatively low (Yu et al., 2015). Cleavage entropies are a measure for the general specificity of a subsite and their combination (Fuchs et al., 2013). Based on the proteomics data of 3,064 peptides from Yu et al. (Yu et al., 2015), which also contains tryptic cleavages, cleavage entropies of 0.962/0.977/0.969/0.966 result for P1′–P4′, in line with the apparent rather low specificity. Roughly 30 diverse substrates would suffice to calculate the specificity for each subsite S4 to S4′ with an uncertainty of 5% (Schauperl et al., 2015). A kinetics study of KLK7 ranked synthetic peptides according to their *k*_cat_/*K*_M_ and corroborated the preference of Tyr and Phe in P1 position, Leu and other hydrophobic residues in P2, whereby P1′ Ser/Arg, P2′ Val/Arg, and P3′ Arg/Ser are most favourable (Oliveira et al., 2015). One has to take into account that the side chains of all basic amino acids possess more extended hydrophobic portions than the acidic ones. Ala, Val or Ile may occupy the same hydrophobic pockets as the Cβ to Cδ atoms of Lys, Arg and His. Thus, a preference for smaller hydrophobic residues and Lys/Arg/His is not contradictory at all.

Apparently, KLK7 exhibits an overall rigid non-prime side region, when comparing the ligand-free structure (PDB code 3BSQ, (Fernández et al., 2008)) with the inhibitor bound ones, e.g. 2QXI (Debela et al., 2007). Also, the molecular dynamics calculations indicate no significant conformational changes that could be interpreted as induced fit upon binding of the reverse backbone substrate and the averaged canonical substrates. There is no evidence for the conformational selection model as well (Vogt & Di Cera, 2013), which might be represented by the minimal form of an essentially single conformational state of KLK7. Thus, most parts of the substrate binding region resemble more the classical lock-and-key model. However, while KLK7 stays comparatively stable, the ligand itself displays an alternative binding pose. The switched binding position does not only provide insights in prime side substrate interactions but can also serve as starting point for drug design efforts. The rare event when the ligand changes its binding orientation could only be observed due to a long sampling time of 10 μs. However, even longer sampling would be necessary to sample all biologically relevant conformations and motions in biomolecules (Henzler-Wildman & Kern, 2007). Recent improvements in simulation speed on specialized hardware allow simulations access to a millisecond time scale (Shaw et al., 2010). Long time scale simulations further the insights on protein-drug interaction, conformational transitions and protein folding (Dror et al., 2012). Alternatively, enhanced sampling techniques cover long time scales at comparatively low computational cost (Abrams & Bussi, 2014; Pierce, Salomon-Ferrer, de Oliveira, McCammon, & Walker, 2012). Relating to drug design efforts extensive sampling of the conformational space is of fundamental importance to find alternative binding modes.

Since KLK7 is overexpressed in human pancreatic ductal adenocarcinomas and cervical cancer cells (Raju, Kaushal, & Haun, 2016), it appears to be an interesting target for specific protease inhibitors (Prassas et al., 2015). However, standard peptides have many drawbacks regarding bioavailability and stability, requiring that the previously known and now revealed positional specificities have to be translated into efficient drug design, e.g. stable retro-inverso peptides (Li et al., 2015). Also, therapeutic antibodies with strong inhibitory effects on serine proteases might a useful approach (Schneider et al., 2012), depending on their applicability for pancreatic and cervical tissue. The extensive interaction of serpins with KLKs, as demonstrated for mutated, sequentially optimized α_1_-antichymotrypsin with KLK2 (Cloutier et al., 2004) and α_1_-antitrypsin with KLK14 (Felber et al., 2006) offers another route to efficient bioavailable drugs as potential anticancer agents, in case the adaptation to KLK7 is feasible.

## Disclosure statement

No potential conflict of interest was reported by the authors.

## Supplemental data

The supplemental data for this paper is available online at https://doi.org/10.1080/07391102.2017.1407674.

## Funding

This work was supported by the Austrian Science Fund FWF [grant number P23051], [grant number P26997] and [grant number P25003-B21 (PG)].

## Supplementary Material

Affinity changes by mutation of the amino acids of the modelled ligand
