# Supplementary material for: An unexpected switch in peptide binding mode: from simulation to substrate specificity
Source: J Biomol Struct Dyn. 2018 Jan 31;36(15):4072–84. doi: 10.1080/07391102.2017.1407674 (PMC6334781; doi:10.1080/07391102.2017.1407674)
Supplement: Affinity changes by mutation of the amino acids of the modelled ligand [file TBSD_A_1407674_SM7723.docx]

**Supporting Information:**

Sequence of the simulated KLK7 protein:

IIDGAPCARGSHPWQVALLSGNQLHCGGVLVNERWVLTAAHCKMNEYTVHLGSDTLGDRR

AQRIKASKSFRHPGYSTQTHVNDLMLVKLNSQARLSSMVKKVRLPSRCEPPGTTCTVSGW

GTTTSPDVTFPSDLMCVDVKLISPQDCTKVYKDLLENSMLCAGIPDSKKNACNGDSGGPL

VCRGTLQGLVSWGTFPCGQPNDPGVYTQVCKFTKWINDTMKKHR

Table S1: Affinity changes by mutation of the amino acids of the modelled ligand at the positions P1’ to P4’. Favourable and highly favourable mutations are highlighted in yellow and red colouring respectively.

|  | dAffinity / kcal/mol | | | |
| --- | --- | --- | --- | --- |
| Residue | P1' | P2' | P3' | P4' |
| A | 0.00 | 0.00 | 0.00 | 0.00 |
| R | -0.70 | -1.53 | -1.33 | -1.31 |
| N | -0.91 | -0.95 | -0.32 | -0.74 |
| D | -1.03 | -0.50 | -0.18 | -0.36 |
| C | -0.37 | 0.02 | -0.15 | -0.35 |
| Q | -0.63 | -0.86 | -0.80 | -0.78 |
| E | -1.45 | -1.05 | -0.59 | -0.68 |
| G | -0.36 | 0.55 | 0.15 | -0.07 |
| H | -1.04 | -1.19 | -1.01 | -0.65 |
| I | -0.47 | -0.61 | -0.31 | -0.67 |
| L | -0.76 | -0.49 | -0.63 | -1.03 |
| K | -0.39 | -0.99 | -0.86 | -1.01 |
| M | -0.78 | -0.94 | -0.51 | -0.33 |
| F | -0.71 | -0.65 | -0.56 | -0.54 |
| P | -0.45 | -0.26 | -0.45 | -0.55 |
| S | -0.58 | 0.01 | 0.02 | -0.21 |
| T | -0.60 | -0.39 | -0.32 | -0.42 |
| W | -0.95 | -1.64 | -0.81 | -1.00 |
| Y | -0.55 | -0.85 | -0.95 | -0.45 |
| V | -0.37 | -0.08 | -0.32 | -0.58 |


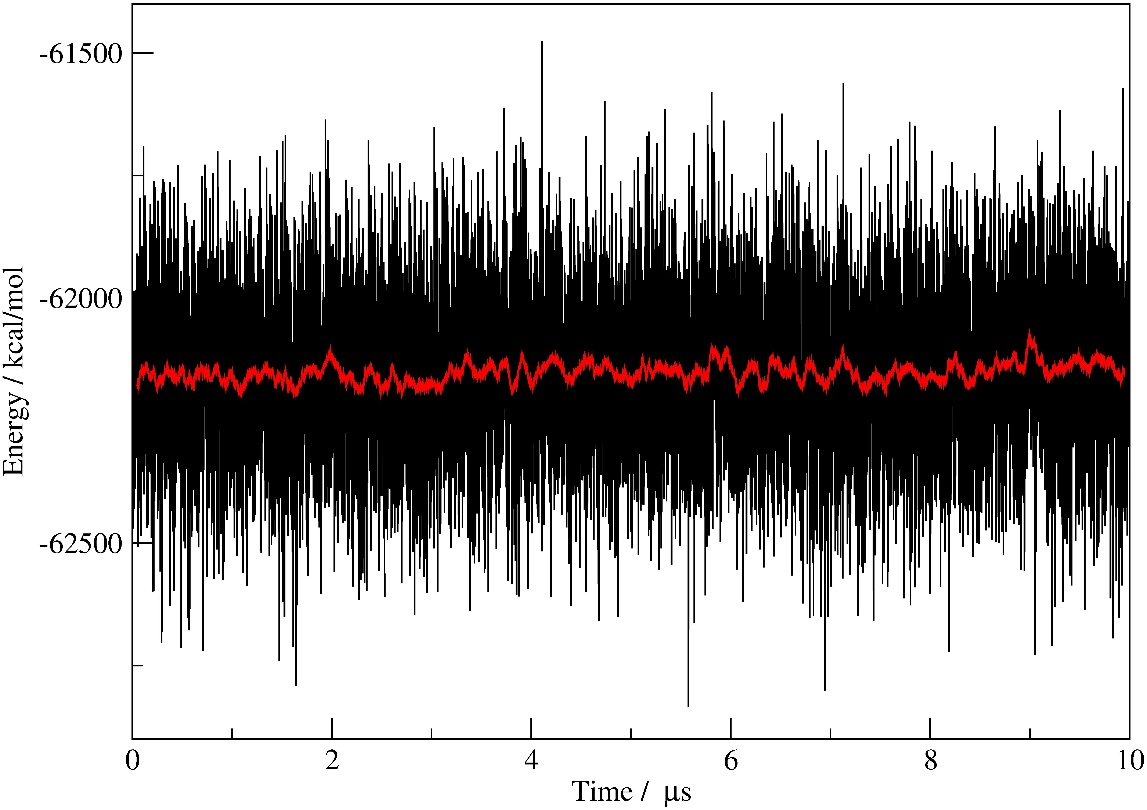


Figure S1: The total energy of the simulated system stays roughly constant during the course of the simulations. The black line marks the actual energy values; the red line represents a running average over 100 ns simulation time.


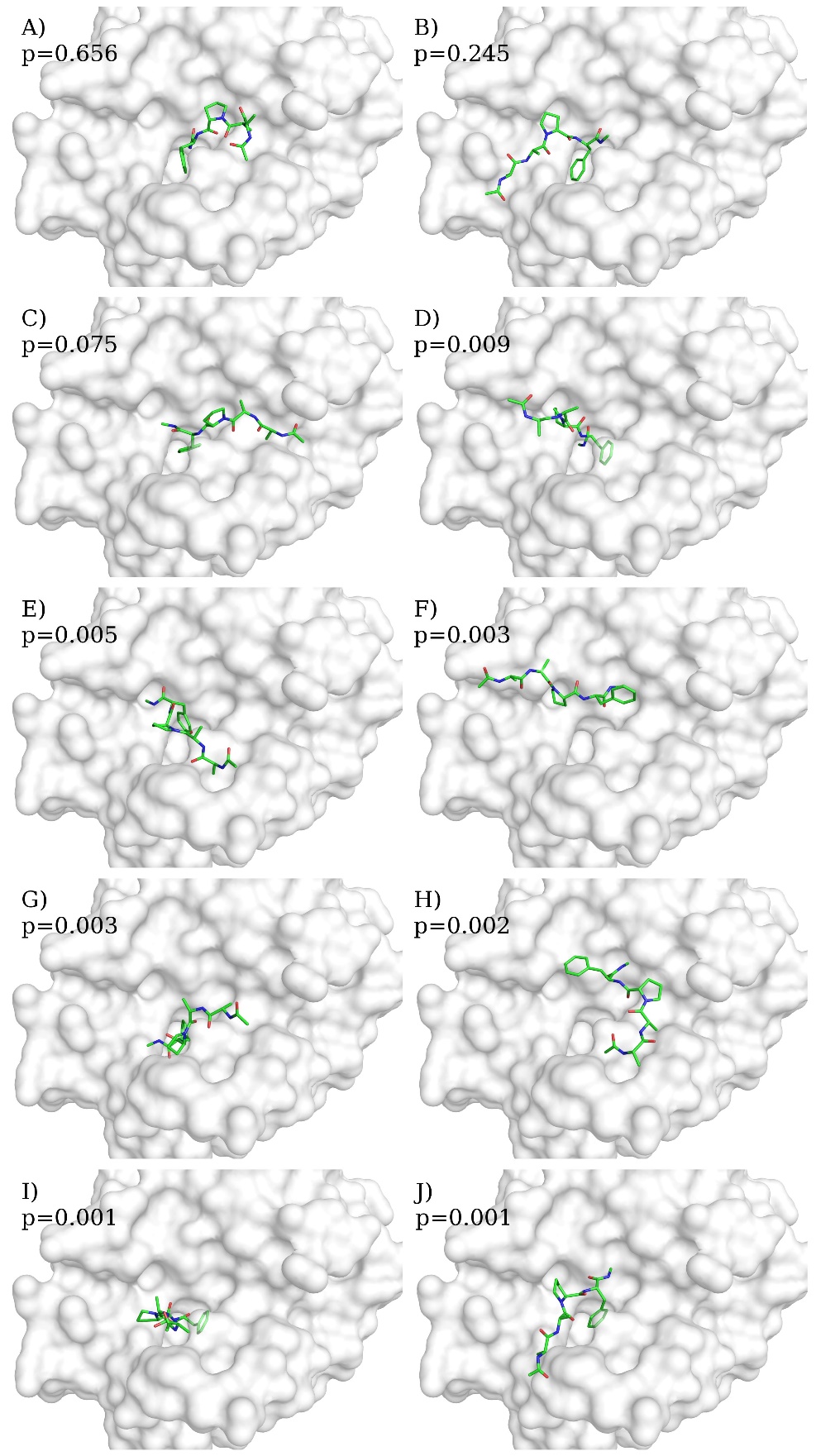


Figure S2: Structures of 10 clusters based on ligand RMSD extracted from the simulation along with their respective occupancies *p*. Only three of the clusters show an occupancy >1%.


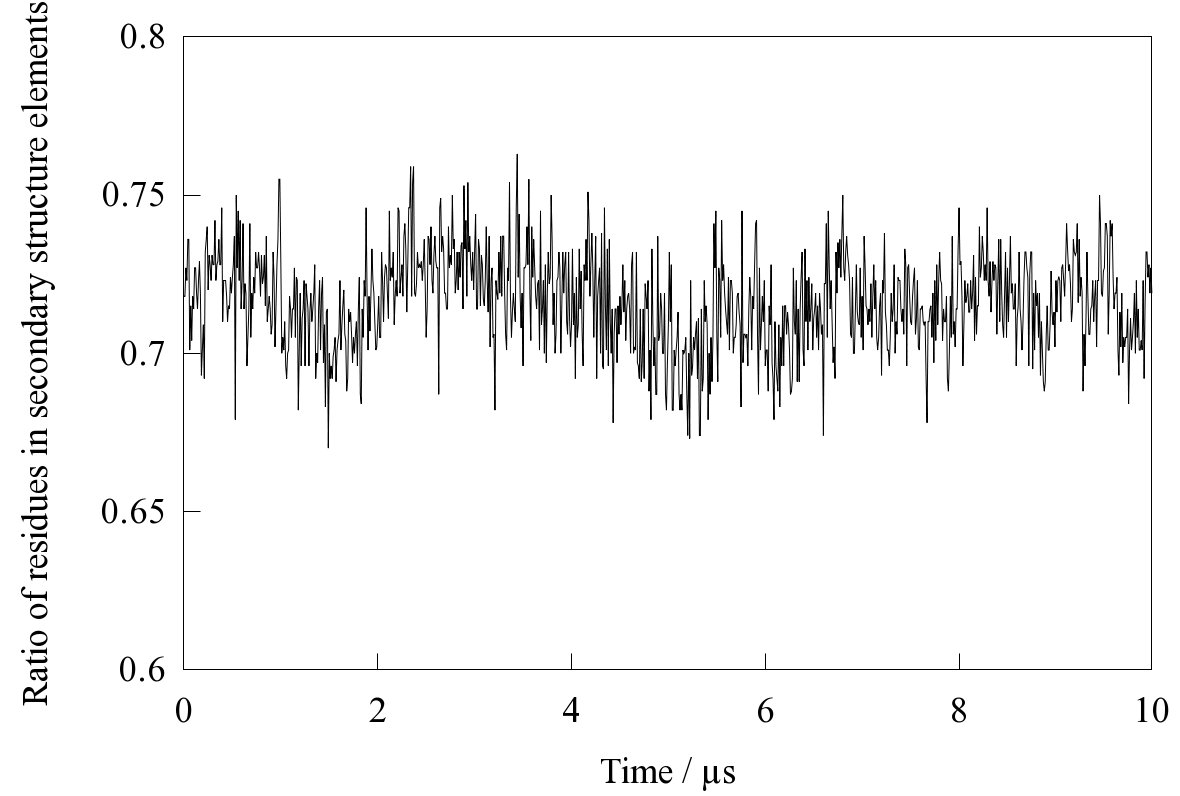


Figure S3: The number of residues that are involved in secondary structure elements according to DSSP assignments shows that no significant unfolding events take place in secondary structure elements during 10 µs simulation time.


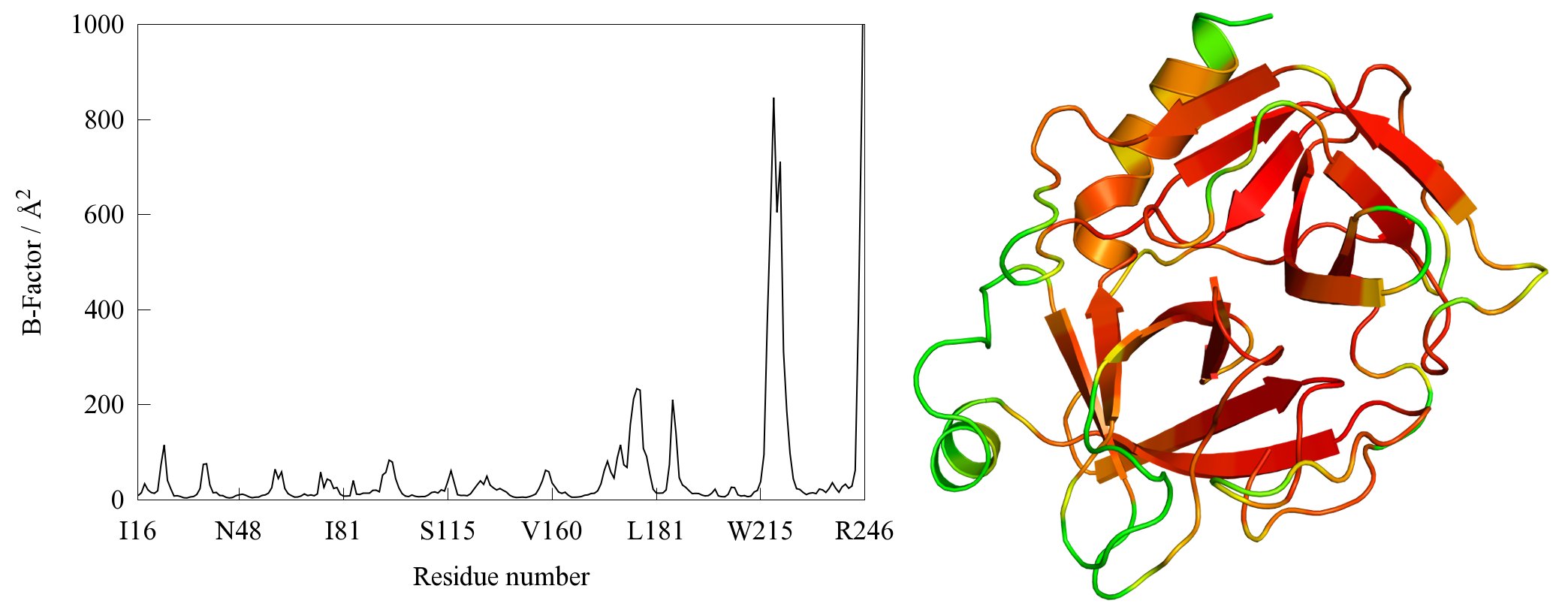


Figure S4: Loop residues and the C-terminus show the highest flexibility. The calculated residue-wise B-factors for Cα atoms are shown on the left. The structure of KLK7 on the right is coloured according to the values to elucidate where the flexible regions are located, with red meaning low and green representing high b-factors.
